# Economic evaluations performed alongside randomized implementation trials in clinical settings: a systematic review

**DOI:** 10.1186/s43058-024-00562-3

**Published:** 2024-03-15

**Authors:** Alayna Carrandi, Amy Grove, Helen Skouteris, Angela Melder, Yanan Hu, Michelle Dever, Alisa Higgins

**Affiliations:** 1 Australian and New Zealand Intensive Care Research Centre (ANZIC-RC), School of Public Health and Preventive Medicine, Monash University, Melbourne, Australia; 2 Warwick Medical School, University of Warwick, Coventry, UK; 3 School of Public Health and Preventive Medicine, Monash University, Melbourne, Australia; 4 Monash Centre for Health Research and Implementation, Clayton, Australia

**Keywords:** Economic evaluation, Clinical trial, Implementation cost, Cost-effectiveness, Implementation economics

## Abstract

**Background:**

Economic evaluations alongside implementation trials compare the outcomes and costs of competing implementation strategies to identify the most efficient strategies. The aims of this systematic review were to investigate how economic evaluations are performed in randomized implementation trials in clinical settings and to assess the quality of these evaluations.

**Methods:**

A systematic literature review was conducted on 23 March 2023 to identify studies that reported on economic evaluations embedded in randomized implementation trials in clinical settings. A systematic search was applied across seven databases, and references of relevant reviews were screened for additional studies. The Drummond Checklist was used to assess the quality and risk of bias of included economic evaluations. Study characteristics and quality assessments were tabulated and described.

**Results:**

Of the 6,550 studies screened for eligibility, 10 met the inclusion criteria. Included studies were published between 1990 and 2022 and from North America, the United Kingdom, Europe, and Africa. Most studies were conducted in the primary and out-patient care setting. Implementation costs included materials, staffing, and training, and the most common approach to collecting implementation costs was obtaining expense and budget reports. Included studies scored medium to high in terms of economic methodological quality.

**Conclusions:**

Economic evidence is particularly useful for healthcare funders and service providers to inform the prioritization of implementation efforts in the context of limited resources and competing demands. The relatively small number of studies identified may be due to lack of guidance on how to conduct economic evaluations alongside implementation trials and the lack of standardized terminology used to describe implementation strategies in clinical research. We discuss these methodological gaps and present recommendations for embedding economic evaluations in implementation trials. First, reporting implementation strategies used in clinical trials and aligning these strategies with implementation outcomes and costs are an important advancement in clinical research. Second, economic evaluations of implementation trials should follow guidelines for standard clinical trial economic evaluations and adopt an appropriate costing and data collection approach. Third, hybrid trial designs are recommended to generate evidence for effective and cost-effective implementation strategies alongside clinical effectiveness and cost-effectiveness.

**Trial registration:**

The review was prospectively registered with PROSPERO (CRD42023410186).

**Supplementary Information:**

The online version contains supplementary material available at 10.1186/s43058-024-00562-3.

Contributions to the literature

•Implementation trials compare the effectiveness of implementation strategies but do not usually compare the cost-effectiveness of these strategies.
•Our review identified 10 economic evaluations performed alongside implementation trials in the clinical setting. This small number of studies may be due to a lack of methodological guidance.
•Economic evidence can influence which interventions are implemented into clinical practice, so it is important to report implementation strategies and related costs in clinical trials.
•Hybrid trial designs that assess both clinical and implementation outcomes are recommended to generate evidence for effective and cost-effective implementation strategies alongside clinical effectiveness and cost-effectiveness.

## Background

The resources available for healthcare must be allocated across a range of competing priorities, so the budget for implementation activities is limited. Despite exponential growth in the field of implementation science in recent years and the increasingly prominent role of economic evidence in health system management, economic evaluations of implementation trials remain both understudied and underreported [1, 2]. Yet, service providers and health care funders would benefit from information that supports or refutes the use of specific implementation strategies as an efficient use of organizational resources [3–7]. This information can inform the prioritization of implementation efforts that maximize patient outcomes within a given level of expenditure. Implementation trials aim to test the effects of implementation strategies on implementation outcomes including the acceptability, adoption, feasibility, fidelity, and sustainability of interventions [5]. Implementation trial designs include types II and III effectiveness-implementation hybrid trials which have a dual focus to evaluate the effects of an evidence-based intervention and assess the effects of implementation strategies [8]. Incorporating economic evaluations appears to be nonstandard practice in assessing implementation strategies [3, 4, 7], which is paradoxical given that they are routinely incorporated in clinical trials.

Economic evaluations compare the costs and consequences of allocating resources among alternative interventions to identify the option that produces the maximum benefit for a given level of expenditure. In the context of implementation trials, economic evaluations compare the outcomes and costs of competing implementation strategies to identify the most efficient strategies and promote uptake and sustained integration of interventions [3, 7, 9]. By not focusing our efforts on understanding the economics of implementation, we risk an inaccurate estimation of the investment required to implement a new intervention and the value of its implementation [10, 11]. Failure to implement interventions is commonly associated with a lack of resources to invest in, and support implementation activities and strategies, or insufficient information regarding costs (i.e. time and resources) of implementing and sustaining new practices [12]. Service providers can equally underestimate the investment required to implement and sustain interventions, whilst also overestimating the investment and pre-emptively choosing not to implement interventions that could benefit patients and the public [9, 11]. Determining efficient implementation strategies is essential to the research translation process in clinical research, as it helps to guide service providers through implementation decision-making processes [3, 4, 10].

Existing systematic and scoping reviews of economic evaluations in implementation science have examined only implementation programmes, public health policy, and quality improvement initiatives [13–15]. None have explored randomized implementation trials in clinical settings. Existing reviews have identified significant heterogeneity in both the implementation costs collected and a practical-knowledge gap on how to conduct economic evaluations across public health and healthcare systems [13–15]. Economic evaluations conducted alongside randomized implementation trials provide an early opportunity to discover the most efficient and effective implementation strategies and to promote the implementation of interventions into clinical practice. Therefore, the aims of this systematic review were to investigate how economic evaluations are performed alongside implementation trials in clinical settings and to assess the quality of these evaluations.

## Methods

### Searches

A systematic literature review was conducted on 23 March 2023 to identify studies that reported on economic evaluations embedded in randomized implementation trials. The primary outcome is the types of economic methods performed within implementation trials. A search strategy was developed and tested in Ovid Medline ® and Epub Ahead of Print, In-Process & Other Non-Indexed Citations using the following three key search parameters: economic methods, randomized trial, and implementation outcomes. The search strategy was then adapted for each of the following databases using the databases’ thesaurus terms: EBM Reviews—Health Technology Assessment, EBM Reviews – NHS Economic Evaluation Database, Embase Classic + Embase, EBSCO – CINAHL Plus, EBSCO – EconLit, and Web of Science – Science Citation Index Expanded. No time or language limiters were applied to the search. Reference lists of eligible studies and review articles were searched for additional relevant studies. Reporting followed the Preferred Reporting Items for Systematic Reviews and Meta-Analyses (PRISMA) [16]. The completed PRISMA checklist is presented in Additional file 1, and the full search strategy is presented in Additional file 2. The review was prospectively registered with PROSPERO (CRD42023410186). A protocol was not published.

### Study inclusion and exclusion criteria

This review aimed to include studies reporting on full economic evaluations alongside randomized implementation trials in the clinical setting (Table 1). Full economic evaluations report on both the costs and consequences of implementation strategies; for example, cost-effectiveness analysis and cost–benefit analysis. Partial economic evaluations that reported on costs without reference to implementation outcomes and modelling studies without trial-based data were excluded.

**Table 1:** Inclusion and exclusion criteria

Inclusion criteria	Exclusion criteria
Randomized assignment of participants	Non-randomized study design
Full economic evaluation • Report on both costs and consequences of implementation strategies	Partial economic evaluation • Report costs without reference to outcomes
Primary study • Trial-based economic evaluation	Study reporting on secondary or non-empirical data • Economic modelling study without trial-based economic evaluation • Systematic review and meta-analysis • Qualitative Study, text and opinion paper, methodology paper, book review, letter, protocol, and conference abstract
Adequately* describe the implementation strategy or strategies being used	Only describes interventions without reference to the implementation strategies employed
Test the effect of implementation strategies on implementation outcomes • Types II and III effectiveness implementation hybrid trials	Report on implementation strategies without testing their effect on implementation outcomes • Type I effectiveness-implementation hybrid trials
Human patients or participants within a clinical setting • Primary, secondary, or tertiary care settings • Digital intervention in conjunction with a health professional	Animal study and human study within a community or non-health sector • School setting • Digital health intervention solely outside of the clinical setting and without linkage with a health professional

^*^According to the Proctor et al. (2013) framework [17]

Included trials had to meet the prerequisites to measuring implementation strategies defined by Proctor et al. [17]. Prerequisites included describing the implementation strategy used, who enacts the strategy, how it is enacted, who is the target of the implementation strategy, when it is used, its dosage, its outcome, and its justification [17]. Examples of implementation strategies included auditing and providing feedback, conducting ongoing training, carrying out local needs assessments, developing educational materials, and using an advisory board or workgroups [18]. Implementation strategy outcomes included adoption, acceptability, uptake, sustainability, and scalability of evidence-based interventions [5]. Other implementation strategy outcomes were trial-specific and depended on the focus of the implementation strategy, such as measures of professional practice improvement, changes in the process of care, adherence to clinical standards, the amount or quality of program or intervention delivery, and required adaptations to the implementation process or strategies based on contextual factors [5].

### Study selection and data extraction strategy

Studies identified in the search were uploaded to Covidence [19], with duplicates automatically removed. Two independent reviewers (MD, YH, and/or AC) screened titles and abstracts, then full-text studies according to the criteria presented in Table 1. Reviewers recorded reasons for exclusion at the full-text stage. Disagreements were resolved through discussion or with an additional independent reviewer (AH and AG). The researchers were blinded to each other’s decisions in both title and abstract screening and full-text review.

**Fig. 1:**
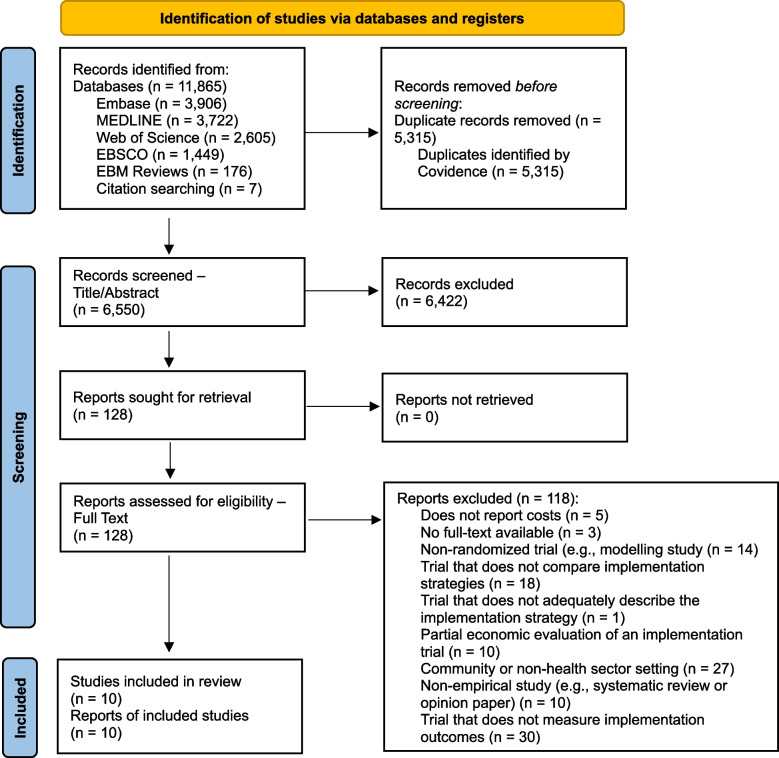
PRISMA flow diagram of eligible studies

The data extraction process was conducted via Covidence [19]. The data extracted included standard bibliographical information (authors, title, journal, and year of publication), study details (study design, setting, and study population), and economic outcomes (perspective, time horizon, discount rate, currency, and measurement and valuation of resources and costs). Data extraction was performed by one reviewer (AC). An additional independent reviewer reviewed the extraction process by referring to the original studies to ensure the accuracy of data extracted (YH).

### Study quality assessment and data synthesis

The Drummond Checklist was used to assess the quality and risk of bias of included economic evaluations [20]. The Drummond Checklist is a comprehensive tool, assessing the methodological quality and risk of bias in the reporting of economic evaluations [20]. It comprises 35 items. Item responses are ‘Yes’, ‘No’, and ‘Not clear’ or, for some methods and analysis items, ‘Not appropriate’. One reviewer (AC) performed the quality and risk of bias assessments, and an additional reviewer (YH) reviewed the assessments by referring to the original studies for accuracy.

Study characteristics, economic methods, and quality assessments were tabulated and described. Additionally, we set out to draw lessons from the narrative body of evidence to provide recommendations for future economic evaluation research. The study authors discussed the important gaps in the literature and commonalities across the studies, such as poor reporting and study design efficiencies. Neither a meta-analysis nor a subgroup analysis was appropriate due to the small number of included studies and no standardized metric across the studies [21].

## Results

Of the 6,550 studies screened for eligibility, 10 met the inclusion criteria (Fig. 1). Many studies excluded at the title and abstract stage did not aim to investigate the cost-effectiveness of implementation strategies, but the cost-effectiveness of interventions. The most common reason for exclusion at the full-text stage was the trial did not measure implementation outcomes (*n* = 30/118; 25%). For example, trials that did not measure implementation outcomes included trials comparing the effectiveness or cost-effectiveness of two interventions without reference to implementation outcomes.

### Study characteristics

Studies were published between 1990 and 2022 with more than 50% published in the past 10 years (Table 2). Most studies were conducted in the primary (*n* = 7; 70% [22–28]) healthcare sector and outpatient settings (*n* = 8; 80% [22–29]). Studies were conducted in the USA (*n* = 4; 40% [22, 27–29]), the UK or Europe (*n* = 3; 30% [25, 26, 30]), and Africa (*n* = 3; 30% [23, 24, 31]). The most common study population was older adults (aged 40 + years) (*n* = 3; 30% [27–29]). Four studies [22, 25, 26, 30] adopted a cluster-randomized approach and randomized units or practices to an implementation strategy (range 7–212 units). The remaining six studies [23, 24, 27–29, 31] randomized an average of 1780 patients (range 83–1655 patients). The most common implementation outcome measured was adoption (*n* = 5; 50% [26–30]).

**Table 2:** Characteristics of 10 implementation trials in a clinical setting with an embedded economic evaluation

Study characteristic	Citation number(s)	Output
Publication year, median (range)		2013 (1990–2022)
Country, *n* (%)		
USA	(22, 27–29)	4 (40%)
UK and Europe	(25, 26, 30)	3 (30%)
Africa	(23, 24, 31)	3 (30%)
Health sector, *n* (%)		
Primary	(22–28)	7 (70%)
Secondary	(29)	1 (10%)
Tertiary	(30, 31)	2 (20%)
Clinical setting, *n* (%)		
Out-patient	(22–29)	8 (80%)
In-patient	(30, 31)	2 (20%)
Study population, *n* (%)		
All patients	(23, 25, 26)	3 (30%)
Older adults (40 + years)	(27–29)	3 (30%)
Birthing people	(30)	1 (10%)
Patients aged 15 + years	(24)	1 (10%)
Children and adolescents	(22, 31)	2 (20%)
Implementation outcomes, *n* (%)		
Quality improvement	(31)	1 (10%)
Adoption	(26–30)	5 (50%)
Penetration	(22–25)	4 (40%)

### Economic evaluation

All included studies examined the incremental costs of implementation relative to the incremental gains in implementation outcomes. Six studies compared the effect of different implementation strategies on the uptake of and adherence to routine screening interventions among primary care [22–24, 27, 28] and secondary care [29] patients. Four studies employed implementation strategies targeted at the healthcare staff to improve the quality of service delivery in hospital [31], the penetration of interventions in hospital [30] and primary care [26], and the adoption of therapy in primary care [25].

Economic perspectives included the healthcare provider (*n* = 2 [22, 31]), societal (*n* = 2 [30, 32]), health payer (*n* = 2 [25, 28]), and healthcare system (*n* = 1 [27]) (Table 3). Time horizons ranged from 3 months [26] to 5 years [22], and only one study used a discount rate [31]. Implementation costs that were collected as a component of the economic evaluation included costs of implementation materials (e.g. supplies, printing, and office space) [22, 23, 28, 29], implementation personnel time (e.g. supervision staff, project staff, and technical assistance) [22–24, 26, 29, 30], and staff training [22–24]. Commonly excluded implementation costs were research-related costs [22, 27–29], such as research staff time and other evaluation costs. Implementation costs were distinct from intervention costs [24, 25, 27]; for example, the staff necessary to deliver the implementation strategy were considered an implementation cost, whereas additional staff necessary to deliver the intervention were considered an intervention cost.

**Table 3:** Methods used to determine the cost-effectiveness of implementation strategies

First author Publication year *Country*	Perspective	Time horizon for implementation, in months	Implementation outcome	Implementation costs collected			Implementation costing approach	Primary ICER for implementation strategy/ies
				Materials	Staff time	Training		
Barasa 2011 [31] *Kenya*	Healthcare provider	18	Quality improvement	✓	✓	✓	Top-down	Cost per percentage gain in mean quality improvement
Costanza 2000 [28] *USA*	Societal and health payer	36	Adoption	✓	✓		Top-down	Cost per additional regular user of the intervention
Edwards 2022 [30] *UK*	Societal	18	Adoption	✓	✓	✓	Top-down	Cost per preterm baby delivered
Bird 1990 [29] *USA*	Not recorded	9	Adoption	✓	✓		Top-down	Cost per additional screening test delivered
Kaner 2003 [26] *UK*	Not recorded	3	Adoption	✓	✓	✓	Top-down	Cost per appropriate intervention delivered
Wagner 2021 [23] *Uganda*	Not recorded	12	Penetration	✓	✓	✓	Top-down	Cost per additional person treated using appropriate method
Meenan 2015 [27] *USA*	Healthcare system	24	Adoption	✓	✓		Bottom-up	Cost per participant current for screening
Claes 2006 [25] *Belgium*	Health payer	6	Penetration	✓	✓	✓	Bottom-up	Cost per day within international normalized ratio range (for patients on anticoagulant therapy)
Nichols 2020 [24] *Malawi*	Not recorded	3	Penetration	✓	✓	✓	Bottom-up	Cost per newly identified positive case; and cost per patient initiated on treatment
Barbosa 2022 [22] *USA*	Healthcare provider	12 and 60	Penetration	✓	✓	✓	Bottom-up	Cost per additional positive screen

*ICER* Incremental cost-effectiveness ratio

Most studies used a top-down approach to collect cost data (*n* = 6; 60% [23, 28–31]; for example, obtaining retrospective expense reports [26, 30, 31] (Table 3). Four studies (30% [22, 24, 25, 27] used bottom-up approaches including activity-based costing and micro-costing where the cost for each individual patient was collected. Bottom-up costing approaches included interviews, questionnaires, and collecting individual patient data on resource use [22, 25, 27]. All incremental cost-effectiveness ratios used trial-specific implementation outcomes in their denominator. Incremental cost-effectiveness ratios are the main output of an economic evaluation and are used to summarize the economic value of an intervention with respect to health effects, such as the cost per quality-adjusted life years gained or cost per years of life lost [33]. For example, Bird et al. [29] assessed the cost-effectiveness of a strategy to promote routine cancer screening among patients by calculating the cost per additional screening test delivered.

### Quality assessment

Overall, the included studies were rated medium (*n* = 2; 20% [28, 29]) to high (*n* = 8; 80% [22–27, 30, 31]) quality (mean = 24.5; standard deviation = 2.67). All studies clearly stated the research question, economic importance of the research question, and rationale for choosing alternative implementation strategies. All studies clearly defined the alternative implementation strategies being compared and the type of economic evaluation used. All studies provided their sources of effectiveness estimates and clearly stated the primary outcome measure for the economic evaluation.

The methods used to value benefits and estimate quantities and unit costs were reported in all studies, and quantities of resource use were reported separately from unit costs. All studies reported the incremental analysis and aggregated and disaggregated outcomes. All studies answered the study question, and the conclusions aligned with the data reported and included appropriate caveats. However, half (*n* = 5; 50% [25, 27–30]) of the studies did not report currency and price data, and seven (*n* = 7; 70% [22, 25–30]) studies did not report details of price adjustments for inflation or currency conversion. Five (*n* = 5; 50% [23, 25, 26, 28, 29]) studies did not explicitly state the time horizons for the economic analysis. The complete quality assessment results are presented in Additional file 3.

## Discussion

The implementation of evidence-based interventions into clinical practice, management, or health policy can be challenging, even when there is strong empirical support for its value in service delivery [12]. Ensuring optimal allocation of the limited resources available for health care has been prioritized among healthcare funders and health service providers worldwide, with economic considerations gaining an increasingly prominent role in the planning, managing, and evaluating health systems [34]. Yet, this service desire for economic evaluation is not matched in the planning and conducting of implementation research. Our systematic review aimed to investigate how economic evaluations are performed within implementation trials in clinical settings and assess the quality of these evaluations. Our review identified 10 economic evaluations performed alongside randomized implementation trials, all of which examined the incremental costs of implementation relative to the incremental gains in implementation outcomes. Included studies spanned a large period (1990–2022) and vast geographical regions, including the USA, UK, Uganda, Malawi, Kenya, and Belgium. Most studies were conducted in the primary and out-patient care setting. Implementation costs included materials, staffing, and training, and the most common approach to collecting implementation costs was obtaining expense and budget reports. Included studies scored medium to high in terms of economic methodological quality.

Among the challenges to conducting economic evaluations alongside implementation trials identified in this review and the broader literature is the lack of empirical evidence for methodology, i.e. absence of standardized processes; inability to differentiate between development, implementation, and intervention costs; and lack of guidance regarding reasonable economic perspectives, cost-effectiveness thresholds, and handling sources of uncertainty [3, 7, 9, 15, 35, 36]. Having conducted this systematic review, we discuss these methodological gaps and present recommendations for embedding economic evaluations in implementation trials. The central recommendations relate to (1) reporting implementation strategies and measuring implementation outcomes, (2) collecting costs using an appropriate costing approach and following guidelines for standard clinical trial economic evaluations, and (3) considering hybrid trial designs to generate evidence for effective and cost-effective implementation alongside clinical effectiveness and cost-effectiveness.

### Report implementation strategies and measure implementation outcomes

The incorporation of economics in implementation trials is neither well-researched nor commonly practiced. We found 10 medium- to high-quality economic evolutions conducted alongside implementation trials in the clinical setting, which is surprising given recent expansions in the field of implementation science [3, 4, 7]. This small number of studies may be due to the lack of comprehensive reporting of implementation strategies within clinical research or variability in the terminology used to describe implementation strategies by clinical researchers across the translational research spectrum [32]. Consequently, implementation strategies are largely underreported or unlabelled, which limits the replication of efficacy and effectiveness results and hinders shared knowledge and language among clinical and implementation researchers [32]. Detailed information on implementation strategies, as outlined by the Proctor et al. framework [17], including information on implementation outcomes, is necessary to establish the effectiveness and cost-effectiveness of implementation strategies. Implementation strategies have costs and can have undesirable consequences, such as inefficiencies and inequities that compromise the accessibility and delivery of health services [4]. Therefore, neglecting to report implementation strategies used in clinical trials as well as identify, measure, and value the costs associated with implementation contributes to the evidence-to-practice gap [12]. There is a risk of thwarting adoption, diminishing reach to individual consumers (especially those experiencing disadvantages and with few resources), furthering poor quality implementation, and hampering sustainability [12]. Reporting implementation strategies used in clinical trials and aligning these strategies with implementation outcomes and costs are an important advancement in clinical research.

### Collect costs using an appropriate costing approach and follow guidelines for standard clinical trial economic evaluations

Estimates of full economic costs and consequences of alternative implementation strategies in clinical settings are also rare [2, 22–31]. This may be because it can be challenging to differentiate between costs related directly to an intervention, costs related to the full implementation process for said intervention, and the costs of any associated changes in healthcare provision and outcomes [2]. Choosing appropriate costs in implementation research is largely context-dependent [1, 3], therefore the standardization of cost data collection and reporting may be difficult to establish. The implementation costs we identified in this review were primarily costs associated with developing and executing implementation strategies, including implementation materials, personnel time, and staff training. Additional implementation costs to those identified in this review include the excess cost of service delivery as uptake or implementation changes, and the opportunity cost to service providers and consumers (e.g. patients) partaking in the implementation activities [4]. Whereas most of the included studies adopted top-down costing approaches, including accessing expense and budget reports, four studies used bottom-up approaches, such as activity-based costing with interviews, questionnaires, and individual patient data.

Adopting a bottom-up approach, such as activity-based costing [37], and collecting qualitative data alongside implementation trials, may be particularly beneficial to health economists, service providers and health care funders who want data that reflect their local context [1, 36]. For example, the Cost of Implementing New Strategies (COINS) is one activity-based costing tool that can be used to map costs associated with implementation activities [9]. Granular cost and outcome data, however, may limit the generalizability of study results to other implementation contexts. A top-down approach may be more feasible in some instances, as these methods use aggregate cost data and allocate costs into various components by some approximate metric (e.g. hospital days) [38]. Although the approach is simple, the cost information may be less accurate in complex organizational settings and instances where human resource costs and overheads comprise a large proportion of the total costs [38]. Researchers must determine whether they want their cost information to reflect as closely as possible the sites under investigation, or whether they want to produce results that could be more generalizable. Researchers should then match their costing approach to their objectives and use methods that optimize precision and accuracy in that situation. Additionally, collecting qualitative data may uncover the value of the long-term sustainment of interventions following initial implementation. In addition to cost reduction, the value of implementing and sustaining interventions may include improving the quality of care for consumers by establishing efficiencies or promoting guideline adherence in service delivery. In the absence of standardized processes for cost data collection and reporting alongside implementation trials, we recommend that economic evaluations of implementation strategies follow guidelines for standard clinical trial economic evaluations [39] and adopt a costing and data collection approach that ensures economic inputs and outputs are contextually relevant for stakeholders [1, 36].

### Consider hybrid trial designs to generate evidence for effective and cost-effective implementation alongside clinical effectiveness and cost-effectiveness

**Fig. 2:**
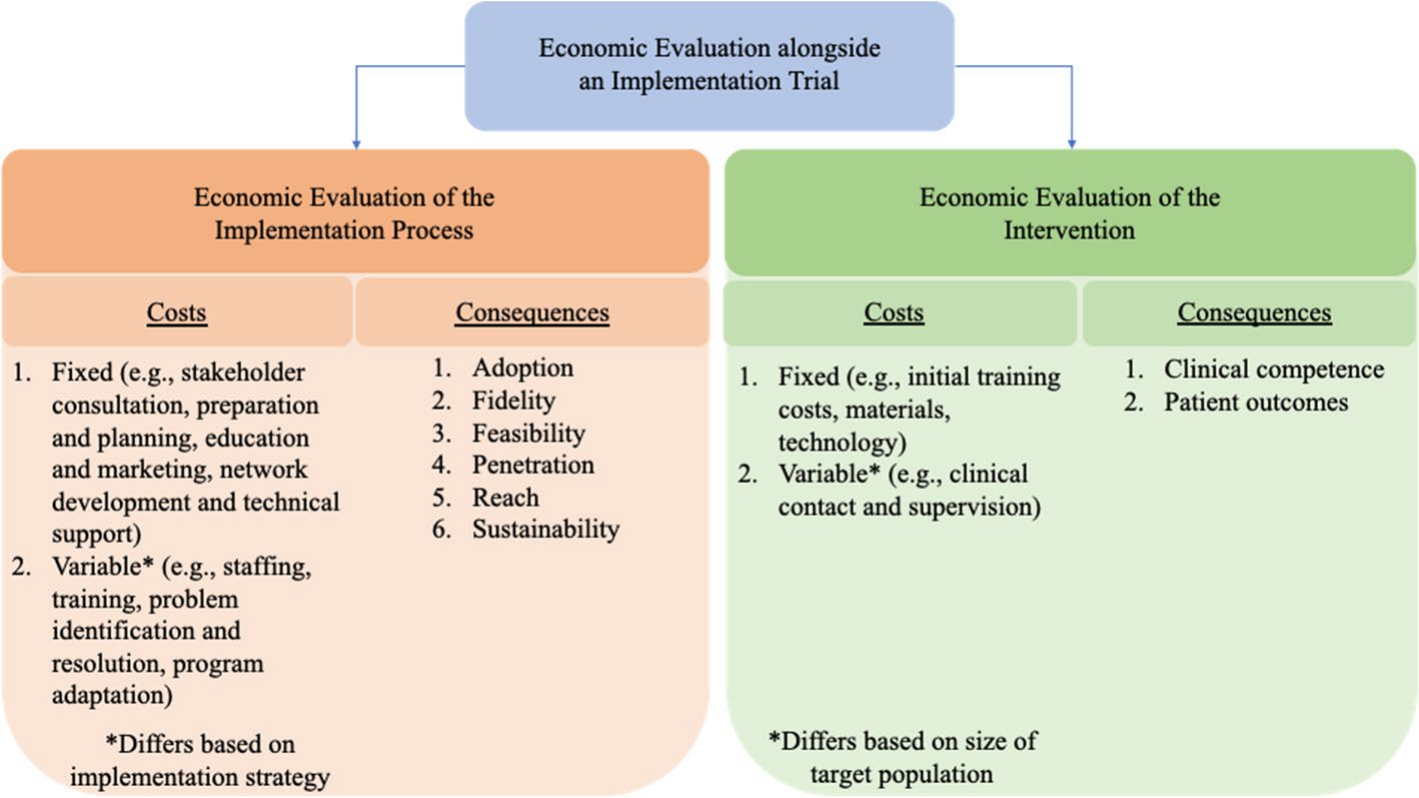
Economic evaluation alongside an implementation trial

Despite the recognized challenges [3, 7, 9, 15, 35, 36], embedding economic evaluations in implementation trials can provide significant benefits to service providers, funders, and consumers [2], particularly in areas of healthcare where creating efficiencies can have a substantial impact. A small proportion of included studies were in the secondary or tertiary and in-patient care settings. Yet, these settings constitute a larger proportion of healthcare spending compared to primary and out-patient care—55% versus 45% in Australia [40], 31% versus 28% in the UK [41], and 31% versus 20% in the USA [42]. The value for money derived from implementation investment and its contribution to the overall efficiency of the health service is informed by the resources directed towards implementation strategies, as well as an assessment of the clinical effectiveness of the intervention [15]. Hybrid trial designs aim to jointly test clinical effectiveness of the intervention while simultaneously evaluating the effectiveness of implementation strategies. We have depicted the components of an economic evaluation in a hybrid implementation trial in Fig. 2. Hybrid trial designs are valuable in terms of accelerating research translation and amplifying the public health impact of clinical research [32] and have been recommended as a way of generating economic evidence alongside implementation evidence [13]. Outcomes derived from economic evaluations in hybrid trial designs could be used by policymakers and researchers to inform strategy selection, while accounting for aspects of feasibility, efficiency, and sustainability. However, adding an economic evaluation increases the implementation trial’s budget, so researchers and local stakeholders must consider the value of this information in their local context. Ensuring effective and cost-effective implementation of interventions is critical for healthcare funders and service providers to improve population and consumer health outcomes and mitigate healthcare costs [15]. We recommend that the generation of evidence for effective and cost-effective implementation should be prioritized alongside evidence for clinical effectiveness and cost-effectiveness to accelerate research translation and amplify the public health impact of clinical research.

### Strengths and limitations

This review followed a rigorous systematic review methodology and reporting followed PRISMA guidelines [16]. Systematic reviews of randomized controlled trials are regarded as the highest levels of evidence in therapeutic areas [43] and included studies were of moderate to high economic methodological quality. Studies also covered vast geographical areas, including both high- and low-and-middle-income countries, and time periods (1990–2022). However, we acknowledge the limitations associated with the geographical and period heterogeneity of studies and the limited number of available studies, which inhibited our ability to perform a meta-analysis. As a result, we focused the results and discussion on the gaps in the evidence base and future opportunities for economic evaluations alongside implementation trials.

We excluded purely modelling studies and only reported on within-trial economic data, given the emerging nature of economic methodology in implementation science and scant economic data in implementation trials. Modelling can be used to project health and economic impacts of implementing an intervention [1, 44], but these models are characterized by assumptions and most likely input values. Generating robust economic data alongside implementation trials will assist in establishing credible economic modelling for implementation. As previously mentioned, implementation strategies and outcomes are largely underreported or mislabelled in the clinical setting [32], so it is possible that some relevant studies may be missing. However, we used a robust searching methodology, and reference lists of eligible studies and review articles were searched for additional relevant studies. Studies were also screened by two independent reviewers, and the data extraction and quality assessments were checked for accuracy.

## Conclusions

Service providers and healthcare funders benefit from information that supports or refutes the use of specific implementation strategies as an efficient use of organizational resources. This systematic review of economic evaluations performed alongside implementation trials in clinical settings identified 10 medium- to high-quality cost-effectiveness studies. Commonly reported implementation costs were categorized into implementation materials, staffing, and training and were commonly collected by obtaining expense and budget reports. Most studies were conducted in the primary and out-patient care setting. Summarizing the existing evidence helped to generate three recommendations for the implementation science field. First, reporting implementation strategies used in clinical trials and aligning these strategies with implementation outcomes and costs can help prioritize implementation efforts in clinical settings. Second, economic evaluations of implementation trials should follow guidelines for standard clinical trial economic evaluations and adopt an appropriate costing approach to ensure data collection is contextually relevant. Third, hybrid trial designs are recommended to generate evidence for effective and cost-effective implementation strategies alongside clinical effectiveness and cost-effectiveness.

### Supplementary Information

**Additional file 1.** Preferred Reporting Items for Systematic Reviews and Meta-Analyses 2020 Checklist [16]. This is a standardized checklist to ensure accurate and high-quality reporting for systematic reviews.

**Additional file 2.** Complete search strategy for economic evaluations embedded in implementation trials. A complete search strategy is presented for each academic database to ensure reproducibility.

**Additional file 3.** Full quality assessment results of included studies using the Drummond Checklist [20]. Quality assessment results are presented for all included studies.

## Data Availability

Data sharing is not applicable to this article as no datasets were generated or analyzed during the current study.

## Abbreviation

PRISMA: Preferred Reporting Items for Systematic Reviews and Meta-Analyses
