# Supplementary material for: Economic evaluations performed alongside randomized implementation trials in clinical settings: a systematic review
Source: Implement Sci Commun. 2024 Mar 15;5:24. doi: 10.1186/s43058-024-00562-3 (PMC10943844; doi:10.1186/s43058-024-00562-3)
Supplement: Supplementary file 2 — Additional file 2. Complete search strategy for economic evaluations embedded in implementation trials. A complete search strategy is presented for each academic database to ensure reproducibility. [file 43058_2024_562_MOESM2_ESM.pdf]

Additional file 2: Complete search strategy for economic evaluations embedded in implementation trials

Name of the database platform: Ovid

Title of the database searched: Medline ® and E-pub Ahead of Print, In-Process, In-Data Review & Other non-indexed citations, daily and versions

Database date range: 1946 to present

Date database last searched: 23 March 2023

Date limits: None

Complete search strategy:

1 cost-benefit analysis/ or cost-effectiveness analysis/ or Economics, Pharmaceutical/

2 ((economic adj2 (evaluation or analys$ or cost$)) or (cost adj2 (effectiv$ or utilit$ or benefi$ or minim$ or efficienc$ or consequenc$)) or pharmacoeconomic$ or (willingness adj2 pay) or (value adj2 money)).ti,ab.

3 1 or 2

4 ((energy or oxygen) adj2 cost).ti,ab.

5 (metabolic adj2 cost).ti,ab.

6 ((energy or oxygen) adj2 expenditure).ti,ab.

7 or/4-6

8 3 not 7

9 (randomized controlled trial or controlled clinical trial).pt.

10 (randomized or randomised or placebo or randomly or "implementation trial" or "effectiveness-implementation trial" or (hybrid adj3 trial)).ab.

11 9 or 10

12 Evidence-based Practice/ or Evidence-Based Medicine/ or Implementation Science/ or Program Evaluation/

13 (acceptability or adoption or appropriateness or feasibility or sustainability or fidelity or penetration or (quality adj2 improvement) or (implementation adj3 cost$)).ti,ab.

14 or/12-13

15 8 and 11 and 14

16 ("community-based" or "community based" or "school-based").ti,ab.

17 15 not 16

18 exp animals/ not humans/

19 exp agriculture/

20 18 or 19

21 17 not 20

22 (letter or editorial or historical article or meta analysis or "review" or "systematic review").pt.

23 21 not 22

Returns: 3722

Name of the database platform: Ovid

Title of the database searched: EBM Reviews – Health Technology Assessment

Database date range: 4th quarter 2016 to present

Date database last searched: 23 March 2023

Date limits: None

Complete search strategy:

1 exp "costs and cost analysis"/ or economics, dental/ or economics, hospital/ or economics, medical/ or economics, nursing/ or economics, pharmaceutical/ or technology assessment, biomedical/ or "health care quality, access, and evaluation"/

2 ((economic$ adj2 evaluation) or (economic$ adj2 cost$) or (economic$ adj2 analys$) or (cost adj2 (effectiv$ or utilit$ or benefi$ or minim$ or efficienc$ or consequenc$)) or pharmacoeconomic$ or (willingness adj2 pay) or (value adj2 money)).ti.

3 1 or 2

4 Evidence-Based Medicine/ or "Diffusion of Innovation"/ or Program Evaluation/

5 (acceptability or adoption or appropriateness or feasibility or sustainability or fidelity or penetration or (quality adj2 improvement) or (implementation adj3 cost$)).ti.

6 4 or 5

7 3 and 6

Returns: 75

Name of the database platform: Ovid

Title of the database searched: EBM Reviews – NHS Economic Evaluation Database

Database date range: 1st quarter 2016 to present

Date database last searched:

Date limits: None

Complete search strategy:

1 (randomized or randomised or placebo or randomly or "implementation trial" or "effectiveness-implementation trial" or (hybrid adj3 trial)).ti.

2 drug therapy.fs.

3 1 or 2

4 Evidence-Based Medicine/ or "Diffusion of Innovation"/ or Program Evaluation/

5 (acceptability or adoption or appropriateness or feasibility or sustainability or fidelity or penetration or (quality adj2 improvement) or (implementation adj3 cost$)).ti.

6 or/4-5

7 3 and 6

Returns: 101

Name of the database platform: Ovid

Title of the database searched: Embase Classic + Embase

Database date range: 1947 to present

Date database last searched: 23 March 2023

Date limits: None

Complete search strategy:

1 exp economic evaluation/ or pharmacoeconomics/

2 ((economic$ adj2 (evaluation or analys$ or cost$)) or (cost adj2 (effectiv$ or utilit$ or benefi$ or minim$ or efficienc$ or consequenc$)) or pharmacoeconomic$ or (willingness adj2 pay) or (value adj2 money)).ti,ab.

3 1 or 2

4 ((energy or oxygen or metabolic) adj2 cost).ti,ab.

5 ((energy or oxygen) adj2 expenditure).ti,ab.

6 4 or 5

7 3 not 6

8 randomized controlled trial/

9 (randomized or randomised or placebo or randomly or "implementation trial" or "effectiveness-implementation trial" or (hybrid adj3 trial)).ab.

10 8 or 9

11 Evidence-based Practice/ or Evidence-Based Medicine/ or Implementation Science/ or Program Evaluation/

12 (acceptability or adoption or appropriateness or feasibility or sustainability or fidelity or penetration or (quality adj2 improvement) or (implementation adj3 cost$)).ti,ab.

13 11 or 12

14 7 and 10 and 13

15 ("community-based" or "community based" or "school-based").ti,ab.

16 14 not 15

17 exp animals/ not humans/

18 exp agriculture/

19 17 or 18

20 16 not 19

21 (letter or editorial or historical article or meta analysis or "review" or "systematic review").pt.

22 20 not 21

Returns: 3906

Name of the database platform: EBSCO

Title of the database searched: CINAHL Plus and Econlit

Database date range: CINAHL Plus (1937 to present); Econlit (1886 to present)

Date database last searched: 23 March 2023

Date limits: None

Complete search strategy:

S1 (MH "Cost Benefit Analysis")

S2 TI ((economic N2 evaluation) or (economic N2 analys##) or (cost N2 (effectiv##### or utilit### or benefi#### or minimi#ation or efficienc### or consequence#)) or pharmacoeconomic# or (willingness N2 pay) or (value N2 money)) OR AB ((economic N2 evaluation) or (economic N2 analys##) or (cost N2 (effectiv##### or utilit### or benefi#### or minimi#ation or efficienc### or consequenc#)) or pharmacoeconomic# or (willingness N2 pay) or (value N2 money))

S3 (S1 OR S2)

S4 TI ((energy or oxygen or metabolic) N2 cost) OR AB ((energy or oxygen or metabolic) N2 cost) OR TI ((energy or oxygen) N2 expenditure) OR AB ((energy or oxygen) N2 expenditure)

S5 S3 NOT S4

S6 (MH "Randomized Controlled Trials")

S7 AB randomized or randomised or placebo or randomly or "implementation trial" or "effectiveness-implementation trial" or (hybrid N3 trial)

S8 S6 OR S7

S9 (MH "Medical Practice, Evidence-Based") OR (MH "Nursing Practice, Evidence-Based") OR (MH "Professional Practice, Research-Based")

S10 (MH "Implementation Science")

S11 (MH "Program Evaluation")

S12 TI (acceptability or adoption or appropriateness or feasibility or sustainability or fidelity or penetration or (quality N2 improvement) or (implementation N3 cost#)) OR AB (acceptability or adoption or appropriateness or feasibility or sustainability or fidelity or penetration or (quality N2 improvement) or (implementation N3 cost#))

S13 S9 OR S10 OR S11 OR S12

S14 S5 AND S8 AND S13

S15 TI ("community-based" or "community based" or "school-based") AND AB ("community-based" or "community based" or "school-based")

S16 (MH "Animals+")

S17 (MH "Agriculture+")

S18 S16 OR S17

S19 S14 NOT S15

S20 S19 NOT S18

S21 S20 NOT (MH "Literature Review+")

Returns: 1456

Name of the database platform: Web of Science

Title of the database searched: Science Citation Index Expanded

Database date range: 1900 to present

Date database last searched: 23 March 2023

Date limits: None

Complete search strategy:

1	TI=((economic NEAR/2 evaluation) or (economic NEAR/2 analys*) or (cost NEAR/2 (effectiv* or utilit* or benefi* or minimi*ation or efficienc* or consequence*)) or pharmacoeconomic* or (willingness NEAR/2 pay) or (value NEAR/2 money)) OR AB=((economic NEAR/2 evaluation) or (economic NEAR/2 analys*) or (cost NEAR/2 (effectiv* or utilit* or benefi* or minimi*ation or efficienc* or consequence*)) or pharmacoeconomic* or (willingness NEAR/2 pay) or (value NEAR/2 money))
2	TI=(((energy or oxygen or metabolic) NEAR/2 cost) OR ((energy or oxygen) NEAR/2 expenditure)) OR AB=(((energy or oxygen or metabolic) NEAR/2 cost) OR ((energy or oxygen) NEAR/2 expenditure))
3	#1 NOT #2
4	AB=(randomized or randomised or placebo or randomly or "implementation trial" or "effectiveness-implementation trial" or (hybrid NEAR/2 trial))
5	TI=(acceptability or adoption or appropriateness or feasibility or sustainability or fidelity or penetration or (quality NEAR/2 improvement) or (implementation NEAR/2 cost*)) OR AB=(acceptability or adoption or appropriateness or feasibility or sustainability or fidelity or penetration or (quality NEAR/2 improvement) or (implementation NEAR/2 cost*))
6	#5 AND #4 AND #3
7	TI=("community-based" or "community based" or "school-based") OR AB=("community-based" or "community based" or "school-based")
8	#6 NOT #7
9	DT=(Editorial Material OR Letter OR Review)
10	#8 NOT #9

Returns: 2605
