# Supplementary material for: Economic evaluations performed alongside randomized implementation trials in clinical settings: a systematic review
Source: Implement Sci Commun. 2024 Mar 15;5:24. doi: 10.1186/s43058-024-00562-3 (PMC10943844; doi:10.1186/s43058-024-00562-3)
Supplement: Supplementary file 3 — Additional file 3. Full quality assessment results of included studies using the Drummond Checklist [20]. Quality assessment results are presented for all included studies. [file 43058_2024_562_MOESM3_ESM.pdf]

Additional file 3: Full quality assessment results of included studies using the Drummond Checklist (20)

Quality assessment item	Included studies									
	Costanza 2000	Meenan 2015	Barasa 2011	Edwards 2022	Bird 1990	Kaner 2003	Claes 2006	Nichols 2020	Wagner 2021	Barbosa 2022
The research question is stated.	Yes	Yes	Yes	Yes	Yes	Yes	Yes	Yes	Yes	Yes
The economic importance of the research question is stated.	Yes	Yes	Yes	Yes	Yes	Yes	Yes	Yes	Yes	Yes
The viewpoint(s) of the analysis are clearly stated and justified.	Yes	Yes	Yes	Yes	No	No	Yes	No	No	Yes
The rationale for choosing alternative programmes or interventions compared is stated.	Yes	Yes	Yes	Yes	Yes	Yes	Yes	Yes	Yes	Yes
The alternatives being compared are clearly described.	Yes	Yes	Yes	Yes	Yes	Yes	Yes	Yes	Yes	Yes
The form of economic evaluation used is stated.	Yes	Yes	Yes	Yes	Yes	Yes	Yes	Yes	Yes	Yes
The choice of form of economic evaluation is justified in relation to the questions addressed.	Yes	Yes	Yes	Yes	Yes	Yes	Yes	Yes	Yes	Yes
The source(s) of effectiveness estimates used are stated.	Yes	Yes	Yes	Yes	Yes	Yes	Yes	Yes	Yes	Yes
Details of the design and results of effectiveness study are given (if based on a single study).	Yes	Yes	Yes	Yes	Yes	Yes	Yes	Yes	Yes	Yes
Details of the methods of synthesis or meta-analysis of estimates are given (if based on a synthesis of a	-	-	-	-	-	-	-	-	-	-

number of effectiveness studies).										
The primary outcome measure(s) for the economic evaluation are clearly stated.	Yes	Yes	Yes	Yes	Yes	Yes	Yes	Yes	Yes	Yes
Methods to value benefits are stated.	Yes	Yes	Yes	Yes	Yes	Yes	Yes	Yes	Yes	Yes
Details of the subjects from whom valuations were obtained were given.	Yes	Yes	Yes	Yes	Yes	Yes	Yes	Yes	Yes	Yes
Productivity changes (if included) are reported separately.	-	-	-	-	-	-	-	-	-	-
The relevance of productivity changes to the study question is discussed.	-	-	-	-	-	-	-	-	-	-
Quantities of resource use are reported separately from their unit costs.	Yes	Yes	Yes	Yes	Yes	Yes	Yes	Yes	Yes	Yes
Methods for the estimation of quantities and unit costs are described.	Yes	Yes	Yes	Yes	Yes	Yes	Yes	Yes	Yes	Yes
Currency and price data are recorded.	No	No	Yes	No	No	Yes	No	Yes	Yes	Yes
Details of currency of price adjustments for inflation or currency conversion are given.	No	No	Yes	No	No	No	No	Yes	Yes	No
Details of any model used are given.	-	-	Yes	Yes	-	-	-	Yes	Yes	-
The choice of model used and the key parameters on which it is based are justified.	-	-	Yes	Yes	-	-	-	Yes	-	-
Time horizon of costs and benefits is stated.	No	Yes	Yes	Yes	No	No	No	Yes	No	Yes

The discount rate(s) is stated.	No	-	Yes	No	-	No	No	-	-	-
The choice of discount rate(s) is justified.	-	-	Yes	No	-	-	-	-	-	-
An explanation is given if costs and benefits are not discounted.	No	No	-	No	-	No	No	No	-	-
Details of statistical tests and confidence intervals are given for stochastic data.	Yes	Yes	Yes	Yes	Yes	Yes	Yes	Yes	Yes	Yes
The approach to sensitivity analysis is given.	-	Yes	Yes	Yes	Yes	Yes	Yes	Yes	-	Yes
The choice of variables for sensitivity analysis is justified.	-	Yes	Yes	Yes	Yes	Yes	Yes	Yes	-	Yes
The ranges over which the variables are varied are justified.	Yes	Yes	Yes	Yes	Yes	Yes	Yes	Yes	Yes	Yes
Relevant alternatives are compared.	Yes	Yes	Yes	Yes	Yes	Yes	Yes	Yes	Yes	Yes
Incremental analysis is reported.	Yes	Yes	Yes	Yes	Yes	Yes	Yes	Yes	Yes	Yes
Major outcomes are presented in a disaggregated as well as aggregated form.	Yes	Yes	Yes	Yes	Yes	Yes	Yes	Yes	Yes	Yes
The answer to the study question is given.	Yes	Yes	Yes	Yes	Yes	Yes	Yes	Yes	Yes	Yes
Conclusions follow from the data reported.	Yes	Yes	Yes	Yes	Yes	Yes	Yes	Yes	Yes	Yes
Conclusions are accompanied by the appropriate caveats.	Yes	Yes	Yes	Yes	Yes	Yes	Yes	Yes	Yes	Yes
**Total score**	**22**	**25**	**31**	**27**	**23**	**24**	**24**	**28**	**24**	**26**
